# Central-line associated bloodstream infections in intensive care units before and after implementation of daily antiseptic bathing with chlorhexidine or octenidine: a post-hoc analysis of a cluster-randomised controlled trial

**DOI:** 10.1186/s13756-023-01260-w

**Published:** 2023-06-03

**Authors:** Luisa A. Denkel, Frank Schwab, Jörg Clausmeyer, Michael Behnke, Jennifer Golembus, Solvy Wolke, Petra Gastmeier, Christine Geffers, the CLIP-ID study group

**Affiliations:** 1 Institute of Hygiene and Environmental Medicine, Charité Universitätsmedizin Berlin, Corporate Member of Freie Universität Berlin, Humboldt-Universität zu Berlin and Berlin Institute of Health, Hindenburgdamm 27, 12203 Berlin, Germany; 2 National Reference Center for the Surveillance of Nosocomial Infections, Charité Universitätsmedizin Berlin, Corporate Member of Freie Universität Berlin, Humboldt-Universität zu Berlin and Berlin Institute of Health, Berlin, Germany

**Keywords:** CLABSI, Antiseptic bathing, Chlorhexidine gluconate, Octenidine dihydrochloride, Post-hoc before-after analysis

## Abstract

**Backgrounds:**

Antiseptic bathing did not reduce central-line (CL) associated bloodstream infection (CLABSI) rates in intensive care units (ICU) according to a recent cluster randomised controlled trial (cRCT). However, this analysis did not consider baseline infection rates. Our post-hoc analysis of this cRCT aimed to use a before-after comparison to examine the effect of daily bathing with chlorhexidine, octenidine or water and soap (control) on ICU-attributable CLABSI rates.

**Methods:**

A post-hoc analysis of a multi-center cRCT was done. ICUs that did not yet perform routine antiseptic bathing were randomly assigned to one of three study groups applying daily bathing with 2% chlorhexidine-impregnated cloths, 0.08% octenidine wash mitts or water and soap (control) for 12 months. Baseline data was assessed 12 months before the intervention started when all ICUs routinely used water and soap. Poisson regression and generalised estimating equation models were applied to identify changes of CLABSI rates per 1000 CL days between intervention and baseline periods in each study group.

**Results:**

The cRCT was conducted in 72 ICUs (24 per study group) including 76,139 patients in the baseline and 76,815 patients in the intervention period. In the chlorhexidine group, incidence density of CLABSI was reduced from 1.48 to 0.90 CLABSI per 1000 CL days comparing baseline versus intervention period (*P* = 0.0085). No reduction was observed in the octenidine group (1.26 versus 1.47 CLABSI per 1000 CL days, *P* = 0.8735) and the control group (1.20 versus 1.17, *P* = 0.3298). Adjusted incidence rate ratios (intervention versus baseline) were 0.63 (95%CI 0.46–0.87, *P* = 0.0172) in the chlorhexidine, 1.17 (95% CI 0.79–1.72, *P* = 0.5111) in the octenidine and 0.98 (95% CI 0.60–1.58, *P* = 0.9190) in the control group. Chlorhexidine bathing reduced CLABSI with gram-positive bacteria, mainly coagulase-negative staphylococci (CoNS).

**Conclusions:**

In this post-hoc analysis of a cRCT, the application of 2% chlorhexidine-impregnated cloths reduced ICU-attributable CLABSI. This preventive effect of chlorhexidine was restricted to CLABSI caused by gram-positive pathogens (CoNS). In contrast, 0.08% octenidine wash mitts did not reduce CLABSI rates in ICUs.

*Trial registration* Registration number DRKS00010475, registration date August 18, 2016.

**Supplementary Information:**

The online version contains supplementary material available at 10.1186/s13756-023-01260-w.

## Background

Pathogens on the body surface of patients are considered a relevant reservoir of nosocomial infections including central-line associated bloodstream infections (CLABSI) [1]. Prevention of CLABSI is crucial, as these healthcare-associated infections (HAI) are associated with increased morbidity, mortality and costs especially in intensive care patients [2, 3]. Decolonisation strategies aim to reduce the number of bacteria on the skin of patients, and subsequently may prevent infections [4].

Several meta-analyses and large-scale randomised controlled trials demonstrate that daily antiseptic bathing with chlorhexidine can significantly reduce bloodstream infections (BSI) and CLABSI in ICUs [5–12]. However, there is still uncertainty whether effectiveness of chlorhexidine-impregnated cloths on CLABSI rates is restricted to ICUs with high infection rates at baseline or certain types of ICUs. Clinical studies that did not show a preventive effect of chlorhexidine-impregnated cloths started with very low rates at baseline or were conducted in non-medical, but surgical ICUs [13, 14].

We conducted the CLIP-ID (Climate and pathogens-impact of decolonisation) study, a cluster-randomised controlled trial (cRCT) in 72 adult intensive care units (ICUs) in Germany and Austria. In the a priori analysis of our cRCT we found no significant preventive effect of 2% chlorhexidine-impregnated cloths or 0.08% octenidine wash mitts on CLABSI rates in ICUs compared to routine care with water and soap (control) [15]. However, our results showed consistent tendencies for the effects of chlorhexidine (risk ratios below 1) and octenidine (risk ratios above 1) on CLABSI rates. Our main analysis had a high likelihood of being underpowered as CLABSI rates were 40% lower than initially expected for our sample size calculation. Adding a pre-test (before period) usually increases the power and precision of statistical tests. Further, a before-after analysis allows considering baseline infection rates and other potential ICU-specific differences between the study groups that might have an impact on CLABSI rates. Thus, in this study, we conducted a post-hoc before-after analysis of our cRCT.

In addition to chlorhexidine-impregnated cloths, we also investigated octenidine wash mitts in our trial, because octenidine dihydrochloride represents a cost-efficient, widely used, easily available antiseptic substance in several European countries [16, 17]. Clinical trials on the effect of antiseptic bathing with octenidine are scarce [18, 19].

Our post-hoc study aimed to analyse the effect of three daily bathing regimes for intensive care patients (2% chlorhexidine-impregnated cloths, 0.08% octenidine wash mitts or continuation of routine care with water and soap as control) on CLABSI rates in a before-after comparison.

## Methods

### Trial design and participants

We performed a post-hoc before-after analysis of the CLIP-ID (climate and pathogens-impact of decolonisation) study. Information on trial design and participants can be found elsewhere [15]. Briefly, we conducted a cluster-randomised controlled trial (cRCT) on 72 ICUs in 68 German and Austrian hospitals. Each ICU that represented one cluster was randomly assigned to one of three study groups. Participating ICUs were required to fulfil the following prerequisites: (1) Continuous surveillance of CLABSI in ICU-KISS during baseline and intervention period, (2) Medical, surgical or interdisciplinary ICUs, (3) CLABSI rates above the median of the respective ICU-KISS reference data within 18 months before recruitment (January 1, 2014–June 30, 2015) or voluntary ICUs with interest in the study and (4) routine bathing regime without antiseptic substances before the intervention period started.

The post-hoc before-after analysis consisted of a 12-months baseline (water and soap) period and a 12-months intervention period (chlorhexidine or octenidine or water and soap). The baseline period lasted from February 1, 2016 to January 31, 2017 and the intervention period from February 1, 2017 to January 31, 2018 in the octenidine and control group. In the chlorhexidine group, baseline (June 1, 2016 to May 31, 2017) and intervention period (June 1, 2017 to May 31, 2018) started four months later due to difficulties in the supply chain.

### Interventions

Each ICU was randomly assigned to one of three bathing regimes: daily patient bathing with non-antiseptic soap and water (control, routine care), daily bathing with 2% chlorhexidine-impregnated cloths (Sage 2% Chlorhexidine Gluconate (CHG)) or 0.08% octenidine disposable wash mitts (Octenisan® by Schülke). Chlorhexidine gluconate is a cationic biguanide compound with strong activity against gram-positive and weaker activity against gram-negative bacteria [20]. Long-term routine use of chlorhexidine may enhance the development of non-susceptibility to this antiseptic substance [21–25]. Octenidine dihydrochloride is a cationic byspiridine that shows activity against gram-positive and gram-negative bacteria [19, 20]. Superiority of octenidine has been demonstrated in vitro in its antiseptic efficacy compared to chlorhexidine [21]. To date, no resistances have been described for octenidine [16, 22].

All ICUs received standard operating protocols (SOP), and education material on infection control and daily bathing procedures (printed material and short videos on step-by-step instructions for bathing and frequently asked questions (FAQs) as downloads) according to their group allocation. Posters and information leaflets were provided to inform patients, patient’s legal representatives, and visitors on participation of the ICU in the trial. All ICUs were encouraged to perform patient bathing at least once a day by trained nurses, to continue all medical interventions including catheter care according to their hygiene plans and standard operating protocols. In the intervention groups, antiseptic products were applied according to manufacturer’s instructions, one (if necessary, more) package(s) of antiseptic products was recommended per patient and day, and on-site trainings were provided by employees of Stryker for chlorhexidine-impregnated cloths and by employees of Schülke for octenidine-impregnated wash mitts.

Details on interventions can be found elsewhere [15]. Briefly, in the routine care group, bathing was continued with any non-antiseptic soap (and water). In the chlorhexidine group, 2% chlorhexidine-impregnated cloths were used below, and non-chlorhexidine containing disposable cloths (provided by Sage Products/Stryker) above the jaw line. Skin care and prophylactic products not provided for treatment were obliged to be compatible with chlorhexidine or dispensed; superficial wounds and devices were allowed to be cleansed by chlorhexidine-impregnated cloths. In the octenidine group, skin care or prophylactic products were allowed to be continued without any restrictions, the face and superficial wounds were allowed to be cleansed by octenidine wash mitts. It was not trained to clean devices with octenidine wash mitts. Compliance monitoring for bathing procedures was done by charting packages of antiseptic products consumed per month and ward and random on-site visits by study personnel in each study group.

Treating physicians and the ICU personnel were responsible for local quality management including on-site implementation of routine care and interventions as well as the management of adverse events. ICUs identified at least two individuals (from the hygiene and/or ICU team) in charge for conducting the CLIP-ID trial on their ward. Adverse events and consumption of decolonisation products were reported monthly to the CLIP-ID study management team. All ICUs were obliged to provide feedback and participate in surveys on infection prevention measures of their wards before and at the end of the intervention period.

The full study protocol including all documentation forms and surveys can be found elsewhere [15].

### Outcomes

This post-hoc before-after analysis of the CLIP-ID cRCT is based on data provided by ICU-KISS, the German national surveillance system for nosocomial infections. Trained nurses and doctors conduced unit-based surveillance by collecting unit-based, anonymous data on number of patients, patient days, days with medical devices (e.g. mechanical ventilation, central line), and HAI including CLABSI. All definitions and details on unit-based surveillance in ICU-KISS can be found in the ICU-KISS protocol [26].

This analysis was done for the primary outcome CLABSI associated with the stay on a participating ICU standardized by central line (CL)-days. Briefly, the definition of CL association to qualify a CLABSI required the following: CL being present at onset of infection (first day with symptoms) or one day before onset of infection for at least the third day. The association to a participating ICU required acquisition of CLABSI on the third day post admission to the ICU at the earliest, while the day of admission was counted as day 1. Further criteria must be met as described elsewhere [15] and in the ICU-KISS protocol [26]:

- Detection of one or more pathogen(s) in one or more blood samples taken for the purpose of diagnosis or treatment by culture-based or non-culture-based methods and the pathogen identified must not be associated to an infection at another site or
- Detection of the same skin bacterium in at least two separate blood samples taken for the purpose of diagnosis or treatment by cultural or non-cultural methods and the pathogen identified must not be associated to an infection on another site and at least one of the following signs or symptoms occurred: fever (> 38 °C), chills, hypotension.

The number of CLABSI with any pathogen was aggregated by the number of CLABSI with gram-positive, gram-negative bacteria, and CLABSI with other causes (fungi or other than fungi, gram-positive or gram-negative pathogens).

Further subgroups analysed were CLABSI with gram-positive bacteria, coagulase-negative staphylococci (CoNS) and gram-negative bacteria.

### Sample size

For this post-hoc before-after analysis, no sample size calculation was performed.

### Randomisation and masking

Details on randomisation and masking can be found elsewhere [15].

### Statistical analysis

All analyses were done with aggregated data on ICU-level. Incidence density of CLABSI was defined as number of newly acquired CLABSI per 1000 CL days. For all incidence rates, exact Poisson 95% confidence intervals (95% CI) were calculated. In the descriptive analysis, numbers with percentages, pooled means and/or medians with interquartile ranges (IQR) were calculated. *P* values were calculated by Chi-square, Mid P exact, Wilcoxon rank sum, Kruskal Wallis or McNemar test.

Differences of incidence densities were tested by unadjusted Poisson regression models. For each outcome, the trial effect was measured by crude incidence rate ratios (IRR) that calculated whether the incidence densities of CLABSI differed significantly between intervention and baseline periods in each study group (chlorhexidine, octenidine and control group). Baseline period included 12 months before the intervention was started for the two intervention groups applying chlorhexidine or octendidine, and the respective first 12 months for the control group continuing daily patient bathing with water and soap.

Crude incidence rate ratios were stratified by CLABSI rates at baseline ≥ or < than 1.0, 0.8 and 0.6 CLABSI per 1000 CL days. The median of all ICUs at baseline is represented by 0.8 CLABSI per 1000 CL days.

In the multivariable analysis, we applied generalised estimating equation (GEE) models based on monthly ward-level aggregated data with negative binomial distribution and accounting for clustering effects [27]. We used generalised estimating equation (GEE) models to compare the outcomes CLABSI with any pathogen, CLABSI with gram-positive bacteria, CLABSI with CoNS and CLABSI with gram-negative bacteria of each study group between the intervention and the baseline period. The GEE offset variable was the logarithmised number of CL days. For epidemiological reasons, the ward-level parameters mechanical ventilation and patients’ length of stay in the current month were determined as possible confounders and considered in all models. The pooled mean for length of stay was defined as number of patient days of ICUs divided by the number of ICU patients, while the pooled mean of mechanical ventilation rate was calculated as number of ventilation days of ICUs divided by the number of patient days of ICUs (multiplied by 100). All parameters added one degree of freedom to the model.

For CLABSI with any pathogen, GEE models were stratified by ICUs with CLABSI rates ≥ or < a certain CLABSI level at baseline. We used three different CLABSI levels for this stratification: 1.0, 0.8 CLABSI and 0.6 CLABSI per 1000 CL days.

*P* values less than 0.05 were considered significant. All analyses were performed using SPSS 25 (IBM SPSS statistics, Somer, NY, USA) and SAS 9.3 (SAS Institute, Cary, NC, USA).

#### Sensitivity analysis

We did sensitivity analyses for the GEE models of the primary outcome “CLABSI with any pathogen” without any further confounders (GEE model crude) and with additional risk factors (GEE model adjusted for additional confounders) to test robustness of our data. Additional confounders for sensitivity analysis included medical ICU (yes/no), antibiotic-coated central lines (yes/no), chlorhexidine-containing dressings (yes/no) and needleless injection ports (yes/no) during the intervention period. Medical ICU was counted as “yes” for all ICUs assigned as medical ICUs, all other ICUs (surgical, medical-surgical, interdisciplinary) were counted as “no”. This data was assessed by ICU-KISS basic data or surveys conducted before and at the end of the intervention period.

Further, all GEE models for CLABSI with any pathogen stratified by CLABSI rates ≥ or < 0.8 CLABSI per 1000 CL days were also tested with the following additional confounders: antibiotic-coated central lines (yes/no), chlorhexidine-containing dressings (yes/no) and needleless injection ports (yes/no) during the intervention period.

## Results

**Fig. 1:**
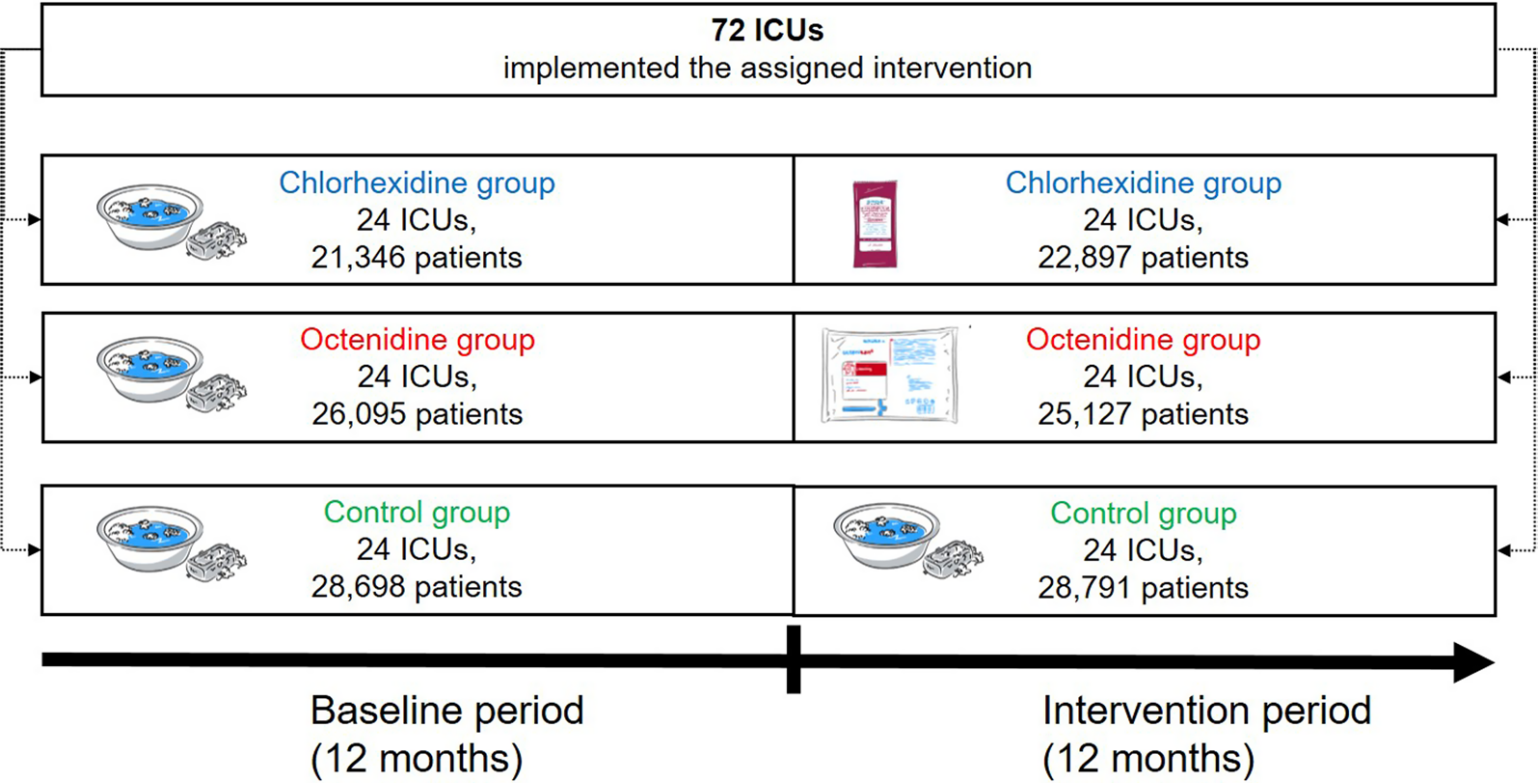
CLIP-ID trial design of the post-hoc before-after analysis

Our post-hoc analysis included 76,139 patients in the baseline and 76,815 patients in the intervention period (Fig. 1 and Table 1). The cRCT was conducted in 72 ICUs (24 ICUs per study group). An overview of our before-after trial design is shown in Fig. 1.

**Table 1:** Characteristics of the study population (ICUs), outcomes and crude incidence rate ratios of intervention versus baseline periods, according to the study group

Parameter	Description	Study group					
		Chlorhexidine		Octenidine		Routine care (control arm)	
		Baseline	Intervention	Baseline	Intervention	Baseline	Intervention
ICUs	N	24	24	24	24	24	24
Patients	N	21,346	22,897	26,095	25,127	28,698	28,791
Patient days	N	75,593	85,135	92,597	90,820	98,726	103,356
CL days	N	46,011	54,305	60,430	58,656	66,670	70,068
Length of stay (days)^b^	Pooled mean	3.5	3.6	3.7	3.4	3.5	3.6
	Median (IQR), *P *value^a^	3.7 (2.6–4.6)	3.6 (2.7–4.2), 0.9179^a^	3.6 (2.7–4.5)	3.6 (2.7–4.5), 1.000^a^	3.3 (2.9–4.2)	3.3 (2.9–4.6), 0.7880^a^
CL use (%)^b^	Pooled mean	60.9	64.6	63.8	67.53	65.3	67.8
	Median (IQR), *P *value^a^	63.4 (55.6–77.5)	67.9 (55.8–77.6), 0.8366^a^	68.4 (45.3–78.5)	64.9 (43.7–77.5), 0.8528^a^	68.5 (60.0–76.8)	65.3 (58.6–79.1), 0.8200^a^
Mechanical ventilation (%)^b^	Pooled mean	34.4	30.8	37.7	35.5	31.9	36.9
	Median (IQR), *P *value^a^	33.2 (24.6–45.9)	33.1 (26.7–47.4), 0.8206^a^	28.2 (17.9–41.3)	30.1 (16.6–39.5), 0.9671^a^	32.2 (24.4–44.9)	34.5 (24.6–48.1), 0.8850^a^
**Outcomes**							
CLABSI with any pathogen	N	68	49	76	86	80	82
	ID/1000 CL days (95% CI)	1.48 (1.15–1.87)	0.90 (0.67–1.19)	1.26 (0.99–1.57)	1.47 (1.17–1.81)	1.20 (0.95–1.49)	1.17 (0.93–1.45)
Compared to baseline in each study group	IRR (95% CI), *P *value	1 = reference	0.61 (0.42–0.88), 0.0085	1 = reference	1.17 (0.86–1.59), 0.3298	1 = reference	0.98 (0.72–1.33), 0.8735
CLABSI with gram-positive bacteria	N	45	28	51	72	52	55
	ID/1000 CL days (95% CI)	0.98 (0.71–1.31)	0.52 (0.34–0.75)	0.84 (0.63–1.11)	1.23 (0.96–1.55)	0.78 (0.58–1.02)	0.79 (0.59–1.02)
Compared to baseline in each study group	IRR (95% CI), *P *value	1 = reference	0.53 (0.33–0.85), 0.0078*	1 = reference	1.45 (1.02–2.08), 0.0407*	1 = reference	1.01 (0.69–1.47), 0.9737
CLABSI with CoNS	N	25	11	31	43	25	26
	ID/1000 CL days (95% CI)	0.54 (0.35–0.80)	0.20 (0.10–0.36)	0.51 (0.35–0.73)	0.73 (0.53–0.98)	0.38 (0.24–0.55)	0.37 (0.24–0.564)
Compared to baseline in each study group	IRR (95% CI), *P *value	1 = reference	0.37 (0.18–0.76), 0.0064*	1 = reference	1.43 (0.90–2.27), 0.1297	1 = reference	0.99 (0.57–1.71), 0.9701
CLABSI with gram-negative bacteria	N	11	12	16	12	14	22
	ID/1000 CL days (95% CI)	0.24 (0.12–0.43)	0.22 (0.11–0.39)	0.27 (0.15–0.43)	0.21 (0.11–0.36)	0.21 (0.12–0.35)	0.31 (0.20–0.48)
Compared to baseline in each study group	IRR (95% CI), *P *value	1 = reference	0.92 (0.41–2.10), 0.8504	1 = reference	0.77 (0.37–1.63), 0.4995	1 = reference	1.50 (0.77–2.92), 0.2393

Baseline period included 12 months before the intervention was started. Parts of  the intervention period have been shown elsewhere [15]. CLABSI, central line associated bloodstream infection. CL, central line. 95% CI, 95% confidence interval. ID, incidence densities per 1000 CL days. IRR, incidence rate ratios calculated by Poisson regression comparing intervention period to baseline period in each study group. CoNS, coagulase-negative staphylococci. ^a^*P* values based on comparison of intervention period to baseline period in each study group. ^b^*P* values were not shown because there were no differences between the three groups during the intervention period. *P values < 0.05 were considered significant

Characteristics of the study population stratified by study groups (chlorhexidine, octenidine, control) and periods (baseline and intervention period) did not differ between baseline and intervention periods in all groups as well as between the groups during the intervention period (Table 1 and Additional file 1: Supplemental table 1).

### CLABSI with any pathogens

**Fig. 2:**
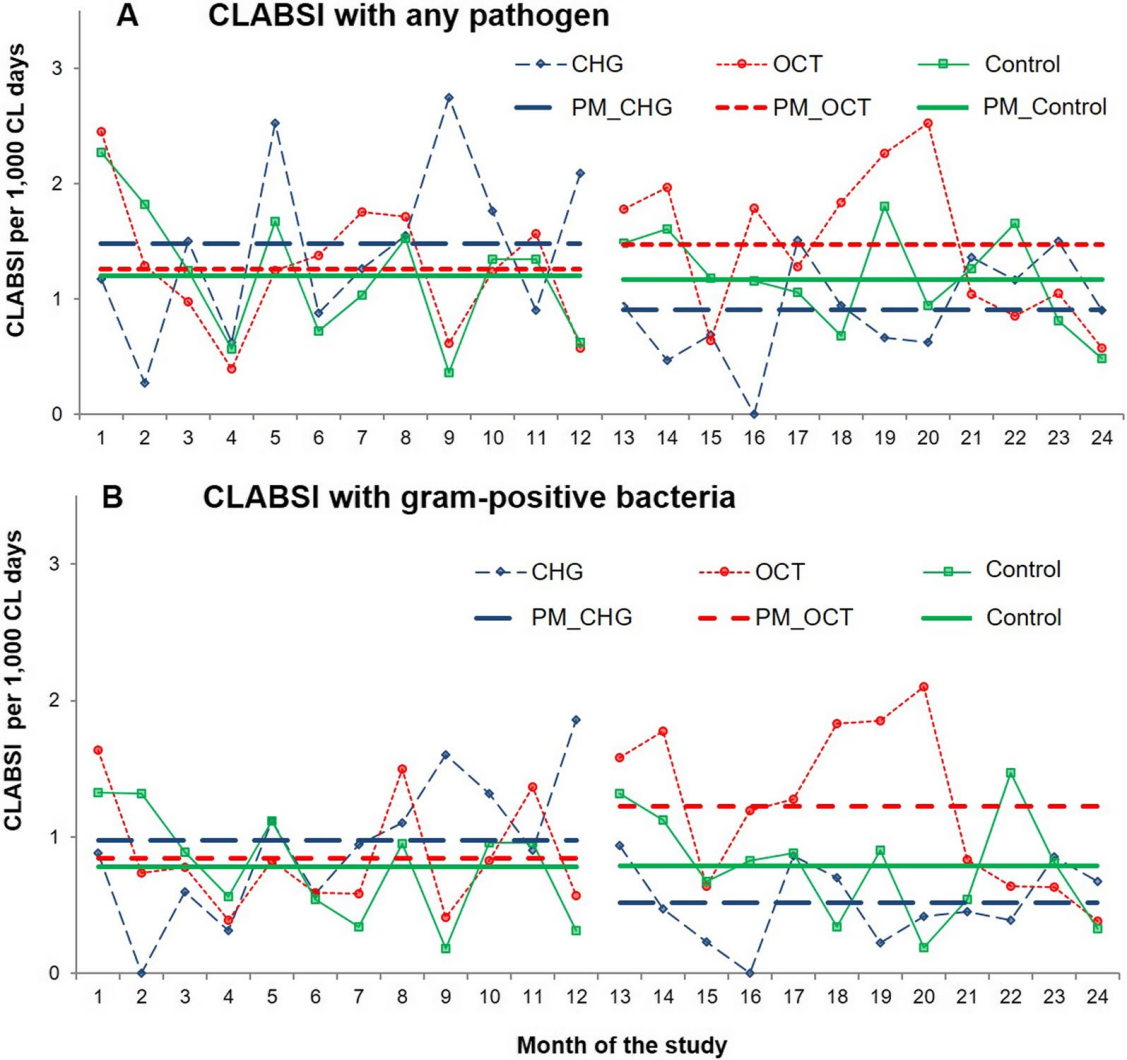
Incidence densities of CLABSI per 1000 CL days with any pathogen (**A**) and gram-positive bacteria (**B**) per study group in baseline and intervention period. Chlorhexidine (CHG, wide dashed line in blue), octenidine (OCT, small dashed line in red) and control group (routine care = water and soap, solid line in green). Months 1–12 represent the baseline, months 13–24 the intervention period. The latter have been shown elsewhere [15]. CLABSI, central line associated bloodstream infections. *CL* central line, *PM* pooled mean

Incidence densities of ICU-associated CLABSI with any pathogen per study group during baseline and intervention periods are visualised in Table 1 and Fig. 2A. In the chlorhexidine group, the CLABSI rate at baseline was noticeably, but non-significantly higher (1.48 CLABSI with any pathogen/1000 CL days) compared to the octenidine (1.26 CLABSI/1000 CL days, *P* = 0.335) and the control group (1.20 CLABSI/1000 CL days, *P* = 0.208).

The crude incidence rate ratios for CLABSI with any pathogen comparing the intervention and baseline periods showed a significant reduction in the chlorhexidine group, but no significant changes in the octenidine and control group (Table 1). In fact, incidence densities of the control group remained stable over the entire observation period (Table 1, Fig. 2).

**Fig. 3:**
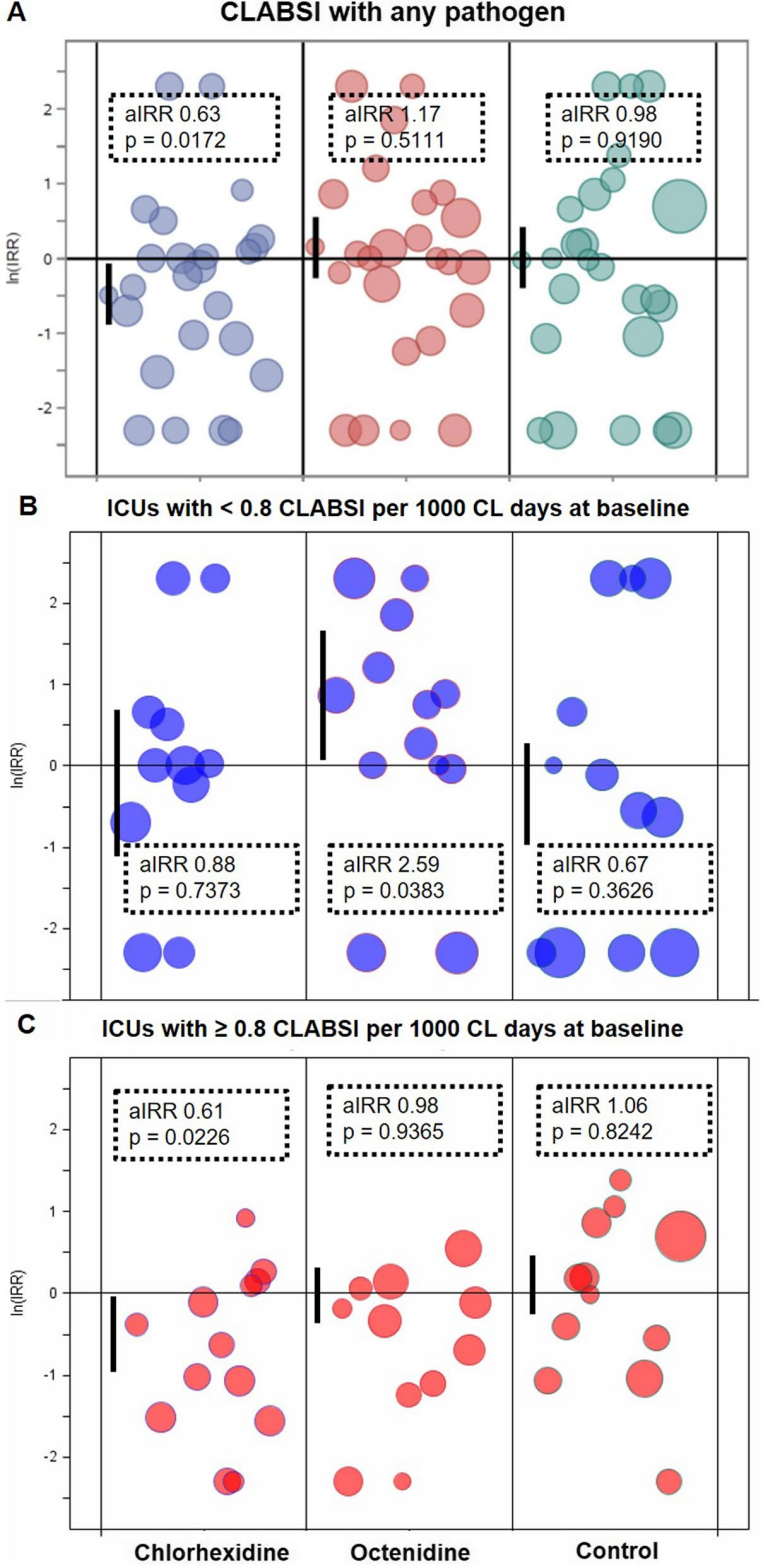
Effect of trial interventions on CLABSI with any pathogen for all ICUs (**A**), for ICUs < 0.8 CLABSI per 1000 CL-days at baseline (**B**) and ICUs with ≥ 0.8 CLABSI per 1000 CL-days (**C**) visualised by study group. Logarithmised incidence rate ratios (ln(IRR)) are depicted for CLABSI with any pathogen. Results are based on unadjusted Poisson regression models (IRR) for single ICUs and adjusted GEE models (aIRR) for study groups (first bubble per group) with 95% confidence intervals (indicated by vertical lines). *P* values were given for type III test. The sizes of bubbles represent the number of CL days from the individual ICU relative to their group effect. For wards that did not observe any case of CLABSI in the baseline or intervention period the IRR was set to 10 (ln(10) = 2.3) and 0.1 (ln(1/10) =  − 2.3), respectively. For the logarithmised incidence rate ratios, equal distances to ZERO (= no effect) in positive or negative directions have the same effect sizes in opposite directions. *aIRR* adjusted incidence rate ratio, *CL* central line (central venous catheter), *ICU* intensive care unit, *IRR* incidence rate ratio

Multivariable analysis adjusting for mechanical ventilation and length of stay confirmed the preventive effect of the daily bathing regime in the chlorhexidine, but not in the octenidine or control group when comparing intervention versus baseline periods for each study group (Table 2, Fig. 3A). The preventive effect of chlorhexidine bathing was found in ICUs with CLABSI rates ≥ 1.0; 0.8 and 0.6 CLABSI per 1000 CL days at baseline, but was not detected in ICUs with CLABSI rates below these levels (Table 3, Additional file 1: Supplemental table 2). Stratified incidence densities are visualised for the level < / ≥ 0.8 CLABSI/1000 CL days as this represents the median CLABSI rate at baseline of all ICUs included (Fig. 3B, C). Antiseptic bathing with octenidine did not reduce ICU-attributable CLABSI neither in ICUs with CLABSI rates ≥ nor < certain CLABSI levels at baseline. In the octenidine group, CLABSI rates even increased in ICUs with < 0.8 CLABSI/1,000 CL days at baseline (Table 3, Fig. 3B, C), but not in ICUs with < 1.0 and 0.6 CLABSI/1000 CL days at baseline (Additional file 1: Supplemental table 2).

**Table 2:** Adjusted incidence rate ratios (aIRR) of intervention versus baseline periods for the outcome CLABSI with any pathogen, CLABSI with gram-positive pathogens, CLABSI with CoNS and CLABSI with gram-negative bacteria according to study group (post-hoc analysis)

Outcome	Chlorhexidine			Octenidine			Routine care (control arm)		
	aIRR (95% CI)	*P *value	*P *value (Type III)	aIRR (95% CI)	*P* value	*P* value (Type III)	aIRR (95% CI)	*P *value	*P *value (Type III)
**CLABSI with any pathogen**									
Baseline period	1 = reference		0.0172*	1 = reference		0.5111	1 = reference		0.9190
Intervention period	0.63 (0.46–0.87)	0.0055		1.17 (0.79–1.72)	0.4394		0.98 (0.60–1.58)	0.9193	
Mechanical ventilation use (per 1%)	1.02 (1.01–1.03)	0.0043	0.0420*	1.01 (0.99–1.02)	0.3808	0.4730	1.02 (0.99–1.04)	0.1505	0.1589
Length of stay (per 1 day)	0.98 (0.87–1.10)	0.6778	0.6712	1.14 (1.06–1.22)	0.0003	0.1134	1.01 (0.89–1.15)	0.8716	0.8754
**CLABSI with gram-positive bacteria**									
Baseline period	1 = reference		0.0580	1 = reference		0.3077	1 = reference		0.9610
Intervention period	0.55 (0.34–0.90)	0.0176		1.46 (0.86–2.50)	0.1651		0.99 (0.55–1.78)	0.9607	
Mechanical ventilation use (per 1%)	1.02 (1.00–1.03)	0.0280	0.0730	1.00 (0.99–1.02)	0.5746	0.6239	1.02 (0.99–1.04)	0.1495	0.1973
Length of stay (per 1 day)	0.94 (0.86–1.04)	0.2440	0.2920	1.11 (0.98–1.25)	0.1172	0.2663	1.04 (0.92–1.18)	0.5404	0.5173
**CLABSI with CoNS**									
Baseline period	1 = reference		0.0359*	1 = reference		0.4452	1 = reference		0.9252
Intervention period	0.38 (0.19–0.74)	0.0043		1.62 (0.63–4.17)	0.3134		1.02 (0.64–1.65)	0.9257	
Mechanical ventilation use (per 1%)	1.03 (1–1.05)	0.0295	0.0990	1.01 (0.99–1.02)	0.2205	0.2804	1.02 (1–1.04)	0.0604	0.1473
Length of stay (per 1 day)	0.95 (0.88–1.03)	0.1786	0.2959	1.05 (0.87–1.27)	0.6020	0.6692	1.01 (0.88–1.17)	0.8531	0.8592
**CLABSI with gram-negative bacteria**									
Baseline period	1 = reference		0.6008	1 = reference		0.4507	1 = reference		0.2151
Intervention period	0.79 (0.34–1.84)	0.5825		0.79 (0.46–1.37)	0.4067		1.5 (0.95–2.37)	0.0792	
Mechanical ventilation use (per 1%)	1.01 (0.98–1.04)	0.4426	0.5020	1 (0.98–1.03)	0.7811	0.7880	1.01 (0.97–1.04)	0.7374	0.7262
Length of stay (per 1 day)	1.07 (0.95–1.21)	0.2633	0.3005	1.28 (1.2–1.36)	< .0001	0.0313	1.05 (0.82–1.36)	0.6844	0.7248

CLABSI, central line associated bloodstream infection. 95% CI, 95% confidence interval. aIRR, adjusted incidence rate ratios. GEE, generalised estimated equation model. Separate GEE models were based on monthly aggregated data, negative binomial distribution, accounted for clustering effects and were calculated with the log number of CL days as offset variable. Parameters considered in all models were mechanical ventilation use and length of stay. * *P* values (type III test) < 0.05 were considered significant

**Table 3:** Adjusted incidence rate ratios (aIRR) of intervention versus baseline periods for the outcome CLABSI with any pathogen stratified by incidence densities (ID) of CLABSI at baseline < or ≥ 0.8 CLABSI/1000 CL-days according to study group (post-hoc analysis)

Outcome/group	Chlorhexidine			Octenidine			Routine care (control arm)		
	aIRR (95% CI)	*P *value	*P *value (Type III)	aIRR (95% CI)	*P* value	*P* value (Type III)	aIRR (95% CI)	*P* value	*P* value (Type III)
CLABSI with any pathogen									
**ICUs with incidence density of CLABSI at baseline < 0.8 CLABSI/1,000 CL days**	n = 11 ICUs			n = 13 ICUs			n = 12 ICUs		
Baseline period	1 = reference			1 = reference			1 = reference		
Intervention period	0.88 (0.42–1.86)	0.7390	0.7373	2.59 (1.61–4.17)	< .0001	0.0383*	0.67 (0.3–1.52)	0.3390	0.3626
Mechanical ventilation use (per 1%)	1.01 (0.99–1.03)	0.4830	0.4275	1.01 (0.98–1.04)	0.3760	0.3927	0.99 (0.96–1.03)	0.7080	0.7800
Length of stay (per 1 day)	0.74 (0.53–1.04)	0.0830	0.1320	1.05 (0.65–1.68)	0.8520	0.8602	1.13 (0.93–1.38)	0.2070	0.3666
** ICUs with incidence density of CLABSI at baseline ≥ 0.8 CLABSI/1000 CL days**	n = 13 ICUs			n = 11 ICUs			n = 12 ICUs		
Baseline period	1 = reference			1 = reference			1 = reference		
Intervention period	0.61 (0.42–0.88)	0.0080	0.0226*	0.98 (0.58–1.64)	0.9380	0.9365	1.06 (0.63–1.78)	0.8210	0.8242
Mechanical ventilation use (per 1%)	1.02 (1–1.03)	0.0160	0.0912	1 (0.99–1.02)	0.8200	0.8405	1.01 (0.99–1.02)	0.4090	0.4278
Length of stay (per 1 day)	0.95 (0.85–1.06)	0.3540	0.3631	1.09 (1.02–1.17)	0.0100	0.2590	1.14 (1.02–1.27)	0.0250	0.0505

*CLABSI* central line associated bloodstream infection. *95% CI* 95% confidence interval, *aIRR* adjusted incidence rate ratios. Separate GEE models were based on monthly aggregated data, negative binomial distribution, accounted for clustering effects and were calculated with the log number of CL days as offset variable. Parameters considered in all models were mechanical ventilation use and length of stay. *P values (type III test) < 0.05 were considered significant

### CLABSI with gram-positive bacteria

For CLABSI with gram-positive bacteria, the incidence densities per study group and period are visualised in Table 1 and Fig. 2B. The crude incidence rate ratios showed a significant reduction of CLABSI with gram-positive bacteria between intervention and baseline period in the chlorhexidine group, a significant increase in the octenidine group, and no change in the control group (Table 1, Fig. 2B). Adjusted incidence rate ratios of CLABSI with gram-positive bacteria, however, found no significant differences between intervention and baseline period in any study group (Table 2).

### CLABSI with CoNS

For CLABSI with CoNS, the crude incidence rate ratio was significantly reduced in the chlorhexidine group, but not in the octenidine or control group (Table 1). These findings were confirmed by adjusted incidence rate ratios (Table 2).

### CLABSI with gram-negative bacteria

In all groups, no changes between intervention and baseline periods were detected for CLABSI with gram-negative bacteria neither in the crude nor in the adjusted analysis (Tables 1, 2).

### Sensitivity analysis

All results of the multivariable analyses for CLABSI with any pathogen were stable in the sensitivity analyses adjusting for additional potential confounders (medical ICU, use of antibiotic-coated CL, chlorhexidine-containing dressings and needless injection ports, Additional file 1: Supplemental table 3).

## Discussion

This is a post-hoc before-after analysis of the first comparative multi-center cRCT that examined antiseptic bathing with chlorhexidine and octenidine for CLABSI prevention in ICUs. In the post-hoc analysis comparing baseline and intervention periods (before-after), chlorhexidine-impregnated cloths significantly reduced ICU-associated CLABSI by about 40%. Decolonisation with chlorhexidine had no effect on CLABSI caused by gram-negative pathogens, but prevented CLABSI caused by gram-positive bacteria, mainly CoNS. In contrast, octenidine wash mitts did not show a preventive effect on CLABSI, neither caused by gram-positive nor gram-negative bacteria. At the same time CLABSI rates remained stable in the control group over the entire observation period.

Our post-hoc analysis confirmed findings of previous studies that showed a preventive effect of chlorhexidine-impregnated cloths on CLABSI rates in ICU patients [7, 11, 12, 28–30]. Most clinical studies that demonstrated a preventive effect of universal decolonisation with chlorhexidine-impregnated cloths had higher infection rates at baseline (≥ 1.7 CLABSI/1000 CL days) [7, 28–35]. In our subgroup analysis, we stratified ICUs according to CLABSI rates ≥ or below < certain CLABSI levels at baseline. These analyses demonstrated that chlorhexidine bathing had a preventive effect in ICUs with CLABSI rates ≥, but not < CLABSI levels of 1.0, 0.8 and 0.6 CLABSI/1000 CL days at baseline.

Octenidine wash mitts did not reduce CLABSI of ICU patients in our study. Thus, we could not confirm the promising results of previous single-center observational trials [18, 19]. One study showed that universal decolonisation of ICU patients with octenidine-containing wash mitts in combination with octenidine nose gel reduced the incidence of primary and secondary ICU-acquired BSI in medical ICUs [18]. We can only speculate about reasons for the lacking preventive effect of octenidine in the CLIP-ID study. Here, we did not use any antiseptic application for the nose, the primary outcome was CLABSI (not including secondary BSI), and the proportion of medical ICUs participating in our trial was below 10%. Further, the protocol for correct application of octenidine wash mitts was not the same as for chlorhexidine. Among other differences, use of any skin care product after daily antiseptic bathing was strictly forbidden in the chlorhexidine, but not in the octenidine group. For octenidine wash mitts, the manufacturer allowed the use of any product 30 s after its application. Further, while chlorhexidine-impregnated cloths contained 2% (20 mg/ml) chlorhexidine, octenidine wash mitts were impregnated with 0.08% octenidine (0.8 mg /ml). Even though, in vitro octenidine is superior in its antiseptic efficacy compared to chlorhexidine, the differences in concentration of these ready-to-use products might be relevant [36].

The reduction of CLABSI in the chlorhexidine group could mainly be attributed to CLABSI with CoNS. At the same time, the increase of CLABSI in the octenidine group was also mainly due to CLABSI with gram-positive bacteria including CoNS. Even though CoNS are considered pathogens of low virulence, catheter-related BSI or bacteremia with CoNS have been associated with severe clinical outcomes including increased duration of hospitalization, therapy related costs and morbidity [37]. Further, CoNS are one of the most frequent causes of CLABSI, also confirmed by our trial. In consequence, prevention of CLABSI with CoNS is clinically relevant and should be taken seriously. CoNS are one of the main sources of blood culture contamination [38]. However, we consider the risk of misclassification as low in our trial as the definition of CLABSI with CoNS required the detection of the same CoNS in at least two separate blood samples and the presence of at least one symptom, e.g. fever or hypotension [26].

Tolerability of chlorhexidine-impregnated cloths and octenidine wash mitts by patients was high and has been reported in detail elsewhere [15]. We performed sensitivity analyses to test the robustness of our data. Results were stable in all sensitivity analyses.

### Strength and limitations

The preventive effect of chlorhexidine-impregnated cloths presented here is based on a post-hoc before-after analysis. The a priori analysis of this cRCT found no significant differences of CLABSI rates between the intervention groups (chlorhexidine and octenidine group) and the control group during the intervention period [15]. However, there is a high likelihood that our main analysis was underpowered as CLABSI rates were 40% lower than initially expected for our sample size calculation [15]. A controlled before-after analysis provides several advantages: First, adding a pre-test (before period) usually increases the power and precision of statistical tests [39]. Further, it allows identifying initial differences between the groups [39]. All important covariates tested (e.g. LOS, CL use, mechanical ventilation) did not differ between the three study groups and two study periods. However, the chlorhexidine group started with non-significant but higher CLABSI rates at baseline compared to the octenidine and control group. The pretest–posttest-control-design allows estimates of treatment effectiveness even when treatment and control group are not equivalent [39]. In consequence, we considered the before-after analysis as most suitable to overcome this non-significant imbalance across the groups. Third, the before-after design allows studying the effect of the intervention at different sublevels of the pre-test. In our stratified analysis, we identified ICUs with CLABSI rates at baseline ≥ 0.6, 0.8 and 1.0 CLABSI/1000 CL days to benefit from chlorhexidine bathing, while ICUs with rates below these levels at baseline did not. This important observation was not available by the post-test only control group design [15]. Convincingly, the control group applying water and soap as routine care did not change during the entire observation period. Thus, the occurrence of systematic or structural changes (e.g. changes of guidelines, medical improvements, etc.) that might have an effect on CLABSI rates in the CLIP-ID wards during the study period is highly unlikely. Before-after analysis is an appropriate approach for randomised controlled trials that has been used before in highly published decolonisation trials [6, 8, 40].

Further, potential confounders including insertion site or usage of ultrasound guidance for central line insertion could have an impact on CLABSI rates [41], but were not assessed by this study. In consequence, they could not be considered in the analyses. Additional limitations of this cluster-randomised decolonisation trial have already been discussed elsewhere [15].

### Generalisability

Most ICUs included in our study had CLABSI rates above the median of the respective ICU-KISS reference data within 18 months before recruitment. This represents a selected study population, and might not be representative for all ICUs. However, it should be notified that “CLABSI rates above the median” referred to the time period between 01/01/2014 and 30/06/2015 (18 months before the recruitment). The baseline period of the study period, however, lasted from February 1, 2016 to January 31, 2017 in the octenidine and control group; and from June 1, 2016 to May 31, 2017 in the chlorhexidine group. Thus, CLABSI rates of ICUs recruited for the CLIP-ID study may have changed until the baseline period started e.g. by the fact of being recruited for the CLIP-ID study.

In our study population, the median CLABSI rates at baseline was 0.8 CLABSI/1000 CL days. Interestingly, this was similar to the ICU-KISS reference data available for all ICUs participating in the surveillance system (2017–2021) [42]. Stratified analyses showed that chlorhexidine bathing had a preventive effect in ICUs with CLABSI rates ≥, but not < certain CLABSI levels at baseline (0.6; 0.8 and 1.0 CLABSI/1000 CL days). Neither ICUs with CLABSI rates ≥ nor < certain CLABSI levels at baseline had a benefit from antiseptic bathing with octenidine.

Type of ICU seems not to have an impact on the effectiveness of antiseptic bathing.

## Conclusion

In this post-hoc analysis of a cRCT the application of 2% chlorhexidine-impregnated cloths had a preventive effect on ICU-attributable CLABSI that was restricted to CLABSI caused by gram-positive pathogens, mainly CoNS. In contrast, 0.08% octenidine wash mitts did not reduce CLABSI rates in ICUs.

## Supplementary Information

**Additional file 1. **Results of the sensitivity analyses are shown in supplemental table 1–3. This includes further characteristics (potential confounders used in GEE models) of the study population (ICUs) in Supplemental table 1; adjusted incidence rate ratios (aIRR) per study group of intervention versus baseline periods for the outcome CLABSI with any pathogen stratified by different CLABSI levels at baseline (< or ≥ 0.6 CLABSI/1000 CL-days and 1.0 CLABSI/1000 CL-days) in Supplemental table 2; and adjusted incidence rate ratios (aIRR) of intervention versus baseline periods for the outcome CLABSI with any pathogen identified by crude GEE models and GEE models adjusted for additional confounders in Supplemental table 3.

## Data Availability

The datasets generated and analysed during the current study are not publicly available as data was collected by the German nosocomial infections surveillance system of intensive care units “ICU-KISS” hosted by the National Reference Center for Surveillance of Nosocomial Infections. The National Reference Center for Surveillance of Nosocomial Infections is committed by a non-disclosure agreement with the hospitals voluntarily participating in ICU-KISS on a confidential basis. It is illegal to publish unaggregated data that enables identification of individual hospitals directly or through a third party. Data sets are available from the National Reference Center for Surveillance of Nosocomial Infections Data Access Committee on reasonable request. Decisions on data requests will consider planned data use, compliance as well as measures for protection of privacy and data security. No individual ward-level results, only aggregated data (e.g. counts, distributions) may be provided. Scientists may send their statement of interest in the trial data to: Data Access Committee Nationales Referenzzentrum (NRZ) für Surveillance von nosokomialen Infektionen at the Institute of Hygiene and Environmental Medicine, Charité-Universitätsmedizin Berlin (nrz@charite.de).

## Abbreviations

aIRR: Adjusted incidence rate ratio
BSI: Bloodstream infection
CHG: Chlorhexidine gluconate
CL: Central line
CLABSI: Central-line associated BSI
CLIP-ID: Climate and pathogens-impact of decolonisation
CoNS: Coagulase-negative staphylococci
95% CI: 95% Confidence interval
cRCT: Cluster randomised controlled trial
FAQ: Frequently asked question
GEE: Generalised estimating equation (model)
HAI: Healthcare-associated infection
ICU: Intensive care unit
ICU-KISS: German national surveillance system for nosocomial infections in intensive care units
ID: Incidence density
IRB: Institutional ethical review board
IRR: Incidence rate ratio
PM: Pooled mean
SOP: Standard operating procedure
