# Supplementary material for: Central-line associated bloodstream infections in intensive care units before and after implementation of daily antiseptic bathing with chlorhexidine or octenidine: a post-hoc analysis of a cluster-randomised controlled trial
Source: Antimicrob Resist Infect Control. 2023 Jun 3;12:55. doi: 10.1186/s13756-023-01260-w (PMC10239202; doi:10.1186/s13756-023-01260-w)
Supplement: Supplementary file 1 — Additional file 1. Results of the sensitivity analyses are shown in supplemental table 1–3. This includes further characteristics (potential confounders used in GEE models) of the study population (ICUs) in Supplemental table 1; adjusted incidence rate ratios (aIRR) per study group of intervention versus baseline periods for the outcome CLABSI with any pathogen stratified by different CLABSI levels at baseline (< or ≥ 0.6 CLABSI/1000 CL-days and 1.0 CLABSI/1000 CL-days) in Supplemental table 2; and adjusted incidence rate ratios (aIRR) of intervention versus baseline periods for the outcome CLABSI with any pathogen identified by crude GEE models and GEE models adjusted for additional confounders in Supplemental table 3. [file 13756_2023_1260_MOESM1_ESM.doc]

**Supplementary material**

**Results**

**Supplemental table 1:** Further characteristics of the study population (ICUs) during the baseline and intervention periods, according to the study group.

| **Parameter** | **Description** | **Study group** |  |  |  |  |  |  |
| --- | --- | --- | --- | --- | --- | --- | --- | --- |
|  |  | **Chlorhexidine** |  | **Octenidine** |  | **Routine care (control arm)** |  | **P-value^b^** |
|  |  | **Baseline** | **Intervention** | **Baseline** | **Intervention** | **Baseline** | **Intervention** |  |
| Type of ICU, all | N (%) | 24 (100.0%) | 24 (100.0%) | 24 (100.0%) | 24 (100.0%) | 24 (100.0%) | 24 (100.0%) |  |
| Surgical | N (%) | 4 (16.7%) | 4 (16.7%) | 5 (20.8%) | 5 (20.8%) | 6 (25.0%) | 6 (25.0%) | 0.9890**^b^** |
| Medical-surgical < 400 beds | N (%) | 13 (54.2%) | 13 (54.2%) | 12 (50.0%) | 12 (50.0%) | 12 (50.0%) | 12 (50.0%) |  |
| Medical-surgical ≥ 400 beds | N (%) | 5 (20.8%) | 5 (20.8%) | 4 (16.7%) | 4 (16.7%) | 4 (16.7%) | 4 (16.7%) |  |
| Medical | N (%) | 2 (8.3%) | 2 (8.3%) | 3 (12.5%) | 3 (12.5%) | 2 (8.3%) | 2 (8.3%) |  |
| Central line care (ward-level) |  |  |  |  |  |  |  |  |
| Antibiotic-coated CL | N (%),p-value^a^ | 4 (16.7%) | 5 (20.8%), 0.4795^a^ | 6 (25.0%) | 7 (29.2%), 0.4795^a^ | 4 (16.7%) | 4 (16.7%), 1.000^a^ | 0.5698**^b^** |
| Chlorhexidine-containing dressings | N (%),p-value^a^ | 8 (33.3%) | 6 (25.0%), 0.2482^a^ | 5 (20.8%) | 5 (20.8%), 1.000^a^ | 8 (33.3%) | 8 (33.3%), 1.000^a^ | 0.6062**^b^** |
| Needleless injection ports | N (%),p-value^a^ | 5 (20.8%) | 8 (33.3%), 0.1336 ^a^ | 9 (37.5%) | 11 (45.8%), 0.2482^a^ | 8 (33.3%) | 7 (29.2%), 0.4795^a^ | 0.4572**^b^** |

CL, central line. CLABSI, central line-associated bloodstream infection. IQR, interquartile range. N, number. N.A. Not applicable as not assessed during baseline period. Data are numbers (n), median (IQR) or pooled mean. The pooled mean (e.g. for length of stay) was calculated as number of patient days of all units divided by the number of patients of all units. Baseline period included 12 months before the intervention was started. IQR, inter quartile range. P-values were calculated by Chi-square, Wilcoxon rank sum or McNemar test. ^a^ P-values comparing baseline and intervention periods within each group.  ^b^ P-values comparing all groups during intervention period.

**Sensitivity analysis**

**Supplemental table 2:** Adjusted incidence rate ratios (aIRR) of intervention versus baseline periods for the outcome CLABSI with any pathogen stratified by high or low incidence densities (ID) of CLABSI at baseline (< or ≥0.6 CLABSI/1000 CL-days and < or ≥ 1.0 CLABSI/1000 CL-days) according to study group (post-hoc analysis).

|  |  | **Chlorhexidine** |  |  | **Octenidine** |  |  | **Routine care**  **(control arm)** |  |  |
| --- | --- | --- | --- | --- | --- | --- | --- | --- | --- | --- |
| **Outcome / Group** |  | **aIRR (95%CI)** | **P-value** | **P-value (Type III)** | **aIRR (95%CI)** | **P-value** | **P-value (Type III)** | **aIRR (95%CI)** | **P-value** | **P-value (Type III)** |
| **CLABSI with any pathogen** |  |  |  |  |  |  |  |  |  |  |
| ICUs with low incidence density of CLABSI at baseline (<0.6 CLABSI / 1000 CL days) |  | n = 8 ICUs |  |  | n = 11 ICUs |  |  | n = 7 ICUs |  |  |
|  | Baseline period | 1=reference |  |  | 1=reference |  |  | 1=reference |  |  |
|  | Intervention period | 1.539 (0.757-3.129) | 0.2337 | 0.3305 | 2.842 (1.623-4.978) | 0.0003 | 0.0589 | 0.903 (0.215-3.783) | 0.8887 | 0.8876 |
|  | Mechanical ventilation use (per 1 %) | 1.028 (1.001-1.056) | 0.0449 | 0.0779 | 1.008 (0.977-1.04) | 0.6208 | 0.6345 | 0.965 (0.946-0.985) | 0.0006 | 0.1593 |
|  | Length of stay (per 1 day) | 0.498 (0.351-0.706) | <.0001 | 0.0339 | 1.138 (0.718-1.804) | 0.5829 | 0.6353 | 1.361 (1.237-1.498) | <0.001 | 0.1161 |
| ICUs with high incidence density of CLABSI at baseline (≥0.6 CLABSI / 1000 CL days) |  | n = 16 ICUs |  |  | n = 13 ICUs |  |  | n = 17 ICUs |  |  |
|  | Baseline period | 1=reference |  |  | 1=reference |  |  | 1=reference |  |  |
|  | Intervention period | 0.594 (0.418-0.844) | 0.0037 | 0.0120 | 0.995 (0.608-1.626) | 0.9826 | 0.9826 | 1.019 (0.66-1.575) | 0.9315 | 0.9321 |
|  | Intervention period | 0.61 (0.42-0.88) | 0.0080 | 0.0226* | 0.98 (0.58-1.64) | 0.9380 | 0.9365 | 1.06 (0.63-1.78) | 0.8210 | 0.8242 |
|  | Mechanical ventilation use (per 1 %) | 1.02 (1-1.03) | 0.0160 | 0.0912 | 1 (0.99-1.02) | 0.8200 | 0.8405 | 1.01 (0.99-1.02) | 0.4090 | 0.4278 |
|  | Length of stay (per 1 day) | 0.95 (0.85-1.06) | 0.3540 | 0.3631 | 1.09 (1.02-1.17) | 0.0100 | 0.2590 | 1.14 (1.02-1.27) | 0.0250 | 0.0505 |
| ICUs with low incidence density of CLABSI at baseline (<1.0 CLABSI / 1000 CL days) |  | n = 13 ICUs |  |  | n = 14 ICUs |  |  | n = 14 ICUs |  |  |
|  | Baseline period | 1=reference |  |  | 1=reference |  |  | 1=reference |  |  |
|  | Intervention period | 0.76 (0.414-1.395) | 0.3765 | 0.3765 | 1.991 (1.039-3.815) | 0.038 | 0.1159 | 0.999 (0.51-1.957) | 0.9976 | 0.9976 |
|  | Mechanical ventilation use (per 1 %) | 1.007 (0.99-1.024) | 0.4210 | 0.4216 | 1.014 (0.984-1.044) | 0.3725 | 0.3847 | 1.018 (0.989-1.048) | 0.2209 | 0.2679 |
|  | Length of stay (per 1 day) | 0.801 (0.629-1.022) | 0.0741 | 0.147 | 1.03 (0.636-1.668) | 0.9054 | 0.9087 | 0.992 (0.806-1.22) | 0.9368 | 0.937 |
| ICUs with high incidence density of CLABSI at baseline (≥1.0 CLABSI / 1000 CL days) |  | n = 11 ICUs |  |  | n = 10 ICUs |  |  | n = 10 ICUs |  |  |
|  | Baseline period | 1=reference |  |  | 1=reference |  |  | 1=reference |  |  |
|  | Intervention period | 0.622 (0.424-0.912) | 0.0152 | 0.0399* | 1.019 (0.629-1.652) | 0.9380 | 0.9394 | 0.958 (0.536-1.71) | 0.8834 | 0.8814 |
|  | Mechanical ventilation use (per 1 %) | 1.013 (0.999-1.026) | 0.0634 | 0.1688 | 1.004 (0.988-1.019) | 0.6440 | 0.7036 | 1.005 (0.987-1.023) | 0.5895 | 0.6053 |
|  | Length of stay (per 1 day) | 0.942 (0.847-1.048) | 0.2722 | 0.3024 | 1.084 (1.014-1.158) | 0.0175 | 0.2659 | 1.182 (1.018-1.374) | 0.0288 | 0.0985 |
|  |  |  |  |  |  |  |  |  |  |  |

CLABSI, central line associated bloodstream infection. 95%CI, 95% confidence interval. aIRR, adjusted incidence rate ratios. GEE, generalised estimated equation model. Separate GEE models were based on monthly aggregated data, negative binomial distribution, accounted for clustering effects and were calculated with the log number of CL days as offset variable. Parameters considered in all models were invasive ventilation use and length of stay. * P-values (type III test) < 0.05 were considered significant.

**Supplemental table 3:** Adjusted incidence rate ratios (aIRR) of intervention versus baseline periods for the outcome CLABSI with any pathogen adjusted for the cluster effect only (GEE model crude) and additional risk factors (GEE model adjusted for additional confounders) including mechanical ventilation, LOS, medical ICU, antibiotic-coated central line (post-hoc analysis), chlorhexidine-impregnated central line dressings, needleless devices.

|  |  | **Chlorhexidine** |  |  | **Octenidine** |  |  | **Routine care**  **(control arm)** |  |  |
| --- | --- | --- | --- | --- | --- | --- | --- | --- | --- | --- |
| **Outcome** |  | **aIRR (95%CI)** | **P-value** | **P-value (Type III)** | **aIRR (95%CI)** | **P-value** | **P-value (Type III)** | **aIRR (95%CI)** | **P-value** | **P-value (Type III)** |
| **CLABSI with any pathogen** |  |  |  |  |  |  |  |  |  |  |
| All |  | n = 24 |  |  | n = 24 |  |  | n = 24 |  |  |
| GEE model crude |  |  |  |  |  |  |  |  |  |  |
|  | Baseline period Intervention period | 1=reference |  |  | 1=reference |  |  | 1=reference |  |  |
|  | Intervention period | 0.642 (0.454-0.909) | 0.0126 | 0.0257* | 1.164 (0.803-1.686) | 0.4222 | 0.5017 | 0.992 (0.589-1.671) | 0.977 | 0.9773 |
| GEE model adjusted for additional confounders |  |  |  |  |  |  |  |  |  |  |
|  | Baseline period | 1=reference |  |  | 1=reference |  |  | 1=reference |  |  |
|  | Intervention period | 0.64 (0.45-0.89) | 0.0092 | 0.0230* | 1.17 (0.79-1.72) | 0.4345 | 0.5101 | 0.98 (0.63-1.55) | 0.9461 | 0.9459 |
|  | Mechanical ventilation use (per 1 %) | 1.02 (1-1.03) | 0.0136 | 0.0578 | 1 (0.99-1.02) | 0.7406 | 0.7594 | 1.01 (1-1.03) | 0.1248 | 0.1262 |
|  | Length of stay (per 1 day) | 0.98 (0.88-1.1) | 0.7466 | 0.7413 | 1.21 (1.07-1.36) | 0.0016 | 0.0972 | 1.09 (0.96-1.24) | 0.1975 | 0.2189 |
|  | Medical ICU | 1.85 (0.72-4.77) | 0.2026 | 0.2294 | 0.62 (0.25-1.56) | 0.3123 | 0.3479 | 1.37 (0.72-2.6) | 0.3315 | 0.4389 |
|  | Antibiotic-coated central line | 1.12 (0.44-2.86) | 0.8188 | 0.8248 | 0.85 (0.34-2.09) | 0.7169 | 0.7266 | 0.45 (0.21-0.97) | 0.0428 | 0.0814 |
|  | Chlorhexidine-containing dressings | 0.49 (0.22-1.11) | 0.0862 | 0.0729 | 0.98 (0.48-2.01) | 0.9544 | 0.9551 | 0.76 (0.42-1.37) | 0.3576 | 0.3206 |
|  | Needleless injection ports | 1.3 (0.52-3.3) | 0.5745 | 0.6227 | 0.46 (0.22-0.99) | 0.0473 | 0.0939 | 0.42 (0.22-0.8) | 0.0083 | 0.0313 |
| ICUs with low incidence density of CLABSI at baseline (<0.8 CLABSI / 1000 CL days) |  | n = 11 |  |  | n = 13 |  |  | n = 12 |  |  |
|  | Baseline period Intervention period | 1=reference |  |  | 1=reference |  |  | 1=reference |  |  |
|  | Intervention period | 0.893 (0.421-1.894) | 0.7684 | 0.7667 | 2.479 (1.511-4.068) | 0.0003 | 0.0451* | 0.684 (0.303-1.545) | 0.3608 | 0.3771 |
|  | Mechanical ventilation use (per 1 %) | 1.004 (0.983-1.026) | 0.6976 | 0.6909 | 1.003 (0.963-1.045) | 0.8809 | 0.8978 | 1.011 (0.981-1.043) | 0.4725 | 0.5157 |
|  | Length of stay (per 1 day) | 0.741 (0.502-1.095) | 0.1329 | 0.1878 | 1.099 (0.729-1.656) | 0.6535 | 0.6907 | 0.967 (0.802-1.166) | 0.7245 | 0.7257 |
|  | Antibiotic-coated central line | 1.247 (0.668-2.327) | 0.4883 | 0.5123 | 0.954 (0.39-2.332) | 0.918 | 0.9213 | 0.428 (0.12-1.527) | 0.191 | 0.1468 |
|  | Chlorhexidine-impregnated central line dressings | 0.828 (0.524-1.311) | 0.4212 | 0.4491 | 2.516 (0.914-6.927) | 0.0741 | 0.1464 | 1.031 (0.486-2.188) | 0.9364 | 0.9374 |
|  | Needleless injection ports | 0.83 (0.505-1.361) | 0.4597 | 0.4534 | 0.596 (0.069-5.15) | 0.6383 | 0.7241 | 1.408 (0.9-2.202) | 0.1338 | 0.2046 |
| ICUs with high incidence density of CLABSI at baseline (≥0.8 CLABSI / 1000 CL days) |  | n = 13 |  |  | n = 11 |  |  | n = 12 |  |  |
|  | Baseline period Intervention period | 1=reference |  |  | 1=reference |  |  | 1=reference |  |  |
|  | Intervention period | 0.616 (0.435-0.873) | 0.0065 | 0.0205* | 0.983 (0.573-1.688) | 0.9508 | 0.9496 | 1.064 (0.62-1.827) | 0.822 | 0.825 |
|  | Mechanical ventilation use (per 1 %) | 1.015 (1.002-1.028) | 0.0202 | 0.0722 | 1.003 (0.993-1.014) | 0.5673 | 0.6408 | 1 (0.986-1.015) | 0.9729 | 0.9744 |
|  | Length of stay (per 1 day) | 0.927 (0.832-1.033) | 0.1703 | 0.2095 | 1.169 (1.029-1.327) | 0.0162 | 0.2161 | 1.204 (1.081-1.341) | 0.0008 | 0.0266* |
|  | Antibiotic-coated central line | 1.839 (1.019-3.32) | 0.0432 | 0.3131 | 0.688 (0.247-1.912) | 0.473 | 0.5088 | 0.595 (0.438-0.809) | 0.0009 | 0.1133 |

CLABSI, central line associated bloodstream infection. 95%CI, 95% confidence interval. aIRR, adjusted incidence rate ratios. GEE, generalised estimated equation model. Separate GEE models were based on monthly aggregated data, negative binomial distribution, accounted for clustering effects and were calculated with the log number of CL days as offset variable. Parameters considered in all models were invasive ventilation use and length of stay. * P-values (type III test) < 0.05 were considered significant.
